# Supplementary material for: Learning curve and surgical outcome of robotic assisted colorectal surgery with ERAS program
Source: Sci Rep. 2022 Nov 29;12:20566. doi: 10.1038/s41598-022-24665-w (PMC9709162; doi:10.1038/s41598-022-24665-w)
Supplement: Supplementary file 1 — Supplementary Information 1. [file 41598_2022_24665_MOESM1_ESM.zip › LSC Raw data-Ñ[▒K/117/H_ñw╝╨░O▒Kñσ.pdf]

正式摘要(電子簽章：0000002112228272)

W52 / W52 ☐英文標題(注意:無英文姓名-請補病人基本檔!) ☐【讀取】憑證

臺中榮民總醫院
出院病歷摘要

轉入號 : 轉出號 :

[redacted]

(8)流水編號	
(9)入院日期	110/02/17 (2021) 直腸外科 W52 - 145
(10)轉科日期	
(11)出院日期	110/02/23 (2021) 住院天數計 6 日

診斷

- 入院 : 1. Adenocarcinoma, moderately differentiated, of rectum, cT3N0, preCCRT CEA 5.91 mg/dL, under CCRT 50 Gy / 25 fr. w/ IGRT to rectal tumor on 2020-10-28~2020-12-01, under xeloda
  2. Thrombosed hemorrhoid s/p total anoplasty on 5/5
- 出院 : 1. Adenocarcinoma, moderately differentiated, of rectum, cT3N0, preCCRT CEA 5.91 mg/dL, under CCRT 50 Gy / 25 fr. w/ IGRT to rectal tumor on 2020-10-28~2020-12-01, under xeloda s/p laparoscopic TME + loop ileostomy on 2021/2/18
  2. Thrombosed hemorrhoid s/p total anoplasty on 5/5

主訴

- For scheduled operation of rectal tumor

病史

- This is a 62 year old patient with history of
  1. Adenocarcinoma, moderately differentiated, of rectum, cT3N0, preCCRT CEA 5.91 mg/dL, under CCRT 50 Gy/25 fr. w/ IGRT to rectal tumor on 2020-10-28~2020-12-01, under xeloda
  2. Thrombosed hemorrhoid s/p total anoplasty on 5/5
  the patient recieved CCRT since 2020/10/28-2020/12/01, now under xeloda. After CCRT, this time the patient was admitted for operation.

體檢發現

- C/W ca of rectum, L/3,fixed........

  bx done
  - General appearance :
    - easy looking , conscious clear , without cardiopulmonary distress
    - Vital signs自動帶入date 體溫(BT) 脈搏(P) 呼吸(R) 血壓(BP) 身高(BH) 體重(BW) BMI
      20210217 36.1℃ 83次/分 18次/分 131/82mmHg 158.4cm 63kg 25.1
    - Vital signs: B.P. 136/94 mmHg , P.R. 108 /min , R.R. 18 /min , B.T. 37 ℃
  - HEENT :
    - Eyes: no anemic conjunctiva, no icteric sclera, normal EOM
    - ENT: no otorrhea, no rhinorrhea, no erythematous mucosa, no injected tonsil, no deformity
  - Neck: supple, freely movable, trachea midline,no deformity, no engorged jugular vein , no palpable thyroid or lymph node enlargement
  - Chest:
    - Inspection: Grossly normal, symmetric and fully expansion, no accessory muscle used, no operation scar, no spider angiomata, no gynecomastia
    - Palpation: no palpable mass or nodule, no subcutaneous emphysema, no tenderness
    - Ausculation: clear breathing sound
  - Heart:
    - Percussion: normal heart size

-
    - Ausculation: regular heart beat, normal S1, S2, no gallop, no murmur
  - Abdomen:
    - Inspection: in
      - flat contour, symetrically, no scar, no stria, no dilated veins, no rashes
    - Ausculation: normoactive bowel sound, no abdominal bruits
    - palpation: soft, no muscle guarding or rigidity, no local tenderness, no rebounding pain, no palpable mass, no palpable pulsation
    - Percussion: no shifting dulllness, no hepatomegaly, no splenomagaly
  -

Peripheral pulsation :	CA	BA	RA	FA	PA	PTA	DPA
Right side :	++	++	++	++	++	++	++/+++
Left side :	++	++	++	++	++	++	++/+++

  - Genito-anus: no tenderness or palpable mass lesion or rectal shelf, normal external genitalia, normal anal tone, brown stool

CRS CONDITION

  -
    - Lung

手術日期及方法

- 手術日期及方法20210218 1. Laparoscopic TME with loop ileostomy
  2. Intraoperative colonoscopy : (-)
  3. High ligation (+) : At IMA root. IMV and left colic artery were also ligated.
  4. EndoGIA (60-4.1) X3 + EEA (33 mm) were applied
  5. 3-O V-Loc was applied for repair the rectal stump site defect
  6. Proximal cut end: > 10 cm, distal cut end: 0.2~0.5 cm
  7. Loop ileostomy was created and fixed over RLQ of abdomen (About 20 cm prior to cecum).
  8. One 12 Fr. penrose drainge tube was placed from LLQ of abdomen and inserted into the pelvic cavity
  9. The colon was not taken down from the splenic flexure site

---

住院治療經過

- After admission, laparoscopic TME + loop ileostomy was done on 2/18. Try water on 2/19 and then bland one diet. The patient could tolerate and stool passage was noted. Under stable condition, the patient was discharge with OPD follow up.

合併症

一般檢查

特殊檢查

病理報告

其它

出院時情況

- 3.改門診治療

出院指示

藥名	劑量	劑量單位	用法	頻次	數量	數量單位	現況	天數	單位
Ultracet tab	1	TAB	PO	Q8HPRN	21	TAB	TH	7	天
DUOdart CAP 0.5/0.4mg	1	CAP	PO	QD	7	CAP	TH	7	天

出院計劃

主治醫師 2062F 蔣鋒帆 / 住院醫師 2332G 林志安

正式摘要(電子簽章：000000211228272)

W52 / W52

doctorRank:B
